# Brain surgery in combination with tyrosine kinase inhibitor and whole brain radiotherapy for epidermal growth factor receptor-mutant non-small-cell lung cancer with brain metastases

**DOI:** 10.1038/s41598-019-53456-z

**Published:** 2019-11-14

**Authors:** Hsin-Hua Lee, Chien-Hung Chen, Hung-Yi Chuang, Yu-Wei Huang, Ming-Yii Huang

**Affiliations:** 1 Program in Environmental and Occupational Medicine, Kaohsiung Medical University and National Health Research Institutes, Kaohsiung, Taiwan; 2 Department of Radiation Oncology, Kaohsiung Medical University Hospital, Kaohsiung, Taiwan; 3 Department of Radiation Oncology, Kaohsiung Municipal Ta-Tung Hospital, Kaohsiung, Taiwan; 4 Department of Occupational and Environmental Medicine, Kaohsiung Medical University Hospital, Kaohsiung Medical University, Kaohsiung, Taiwan; 5 Faculty of Department of Public Health, College of Health Science, Kaohsiung Medical University, Kaohsiung, Taiwan; 6 Department of Radiation Oncology, Kaohsiung Chang Gung Memorial Hospital, Kaohsiung, Taiwan; 7 Department of Radiation Oncology, Faculty of Medicine, College of Medicine, Kaohsiung Medical University, Kaohsiung, Taiwan; 8 Drug Development and Value Creation Research Center, Kaohsiung Medical University, Kaohsiung, Taiwan; 9 Center for Cancer Research, Kaohsiung Medical University, Kaohsiung, Taiwan

**Keywords:** Outcomes research, Non-small-cell lung cancer

## Abstract

The role of brain surgery (BS) on the survival of patients with non-small-cell lung cancer (NSCLC) and brain metastases (BM), particularly those with epidermal growth factor receptor (EGFR) mutations under tyrosine kinase inhibitors (TKIs) is yet to be defined. We aimed to investigate whether BS could improve the survival of patients in addition to the combination of TKIs and whole brain radiotherapy (WBRT). A cohort of 1394 NSCLC patients between 2011 and 2016 was retrospectively studied. One hundred patients with BM receiving TKI + RT were enrolled. Forty patients (40%) received TKI + BS + RT, and 60 patients (60%) received TKI + RT. Survival time was calculated from the date of BM diagnoses to the date of death or last follow-up. With a median follow-up of 25.6 months (95% CI, 18.6–35.7), the median survival after BM was 18.2 months (95% CI, 10.8 to 27.4) in the TKI + BS + RT group and 11.8 months (95% CI, 5.2 to18) in the TKI + RT group. Cox proportional hazards regression model for the patients with the largest BM over 1 cm showed that TKI + BS + RT group was associated with improved survival relative to TKI + RT group (HR, 0.49; 95% CI, 0.29 to 0.83; *P* = 0.008). BS adds significant survival benefits in addition to TKIs and WBRT, especially for patients with EGFR-mutant NSCLC and the largest BM over 1 cm.

## Introduction

Thirty years ago, the median overall survival (OS) after a diagnosis of brain metastasis (BM) for patients with lung adenocarcinoma was 73 days^1^. Lung cancer remains lethal in all nations: 5-year OS is below 20% everywhere in Europe, in the range 15–19% in North America, and as low as 7–9% in Mongolia and Thailand^2^. The latest U.S. study utilized the National Cancer Data Base to identify 457481 patients with non-small-cell lung cancer (NSCLC) diagnosed between 2010 and 2012. The median and 1-, 2-, and 3-year OS for these patients with BM were 6 months and 29.9%, 14.3%, and 8.4% respectively^3^.

In the epoch of target therapies, screening for specific mutations to guide treatment is necessary. The predictive factors for epidermal growth factor receptor (EGFR) mutations are the female sex, never-smoker status, adenocarcinoma histology, and East Asian racial origin^4,5^. However, a U.S. study of 2142 patients with stage I to IV NSCLC found that EGFR mutations in tumors from ever smokers represented 40% of all mutations detected and those from men represented 31%^6^. A prospective study of 1482 patients confirmed the EGFR mutation frequency of 51.4% overall in tumors from Asian patients with adenocarcinoma^7^. Now it is mandatory to detect EGFR mutation prior to treatment. EGFR tyrosine kinase inhibitors (TKIs) have shown a response rate of 70–80% with improved progression-free survival (PFS) and OS than those obtained with standard chemotherapy in patients harboring EGFR mutations^8^. The use of any first- or second-generation EGFR-TKIs alone for the treatment of intracranial involvement in patients with EGFR mutant-positive lung adenocarcinoma showed a favorable cerebral response rate of more than 50%^9,10^.

The optimal treatment of BM is debatable. Classic therapeutic options include local therapies such as whole-brain radiation therapy (WBRT), stereotactic radiosurgery and surgical resection, EGFR-TKIs, and chemotherapy. Systemic chemotherapy is considered futile in the treatment of intracranial involvement due to the blood-brain barrier (BBB), which includes efflux pumps on brain capillaries^11^. With the evolution of sophisticated radiation technology, most clinicians integrate radiotherapy (RT) into the comprehensive treatment of patients under TKIs in order to maximize the therapeutic effects^12,13^. The life expectancy in EGFR-mutant patients has been significantly prolonged^14–16^. Because of an aging population and advances in the treatment of NSCLC, patients are living longer and are more likely to experience distant metastases. A recent Taiwanese study reported that EGFR mutation was a predictor for subsequent BM^17^. Patients with EGFR-mutated NSCLC have a higher cumulative incidence of BM^18^. They can be surgical candidates because they have already demonstrated the proclivity to longevity despite their cancer diagnoses.

Some may consider novel targeted agents (TKIs: gefitinib, erlotinib, afatinib, icotinib, and osimertinib) are potential alternatives to surgical resection since the survival benefit of surgery seems limited to the subgroup of patients with controlled systemic disease and good performance status^19^. However, improvement in neurosurgical techniques such as microneurosurgery, use of neuronavigation, intraoperative imaging, and cortical and subcortical mapping, along with concurrent progress in neuroanesthesia, has substantially decreased surgical morbidity and mortality^20^. Surgical resection remains an important tool for treating BM from NSCLC, particularly in patients with one large or symptomatic lesion^21^. Patients who received TKIs after a diagnosis of stage IIIB or IV lung cancer and WBRT were reimbursed by the Bureau of National Health Insurance of Taiwan^22^. Herein, we analyzed the factors affecting the prognosis for the patients with EGFR-mutant NSCLC BM under both TKIs and WBRT. The efficacy of brain surgery (BS) will be scrutinized in this research article.

## Methods

### Ethics approval statement

The present study (KMUHIRB-E(II)-20180185) was approved and conducted under compliance of the Institutional Review Board (IRB) regulations of Kaohsiung Medical University Hospital. All patients provided written informed consent prior to RT and/or BS. Patient information was anonymized and de-identified before analysis. All data were analyzed anonymously and retrospectively.

### Patients and treatment

We sorted 1394 NSCLC patients in the data base of a tertiary university hospital and retrospectively recruited one hundred consecutive patients with pathologically proven lung adenocarcinoma who had received both WBRT and TKIs between January 1, 2011 and June 14, 2016. Their BM was diagnosed by either brain imaging or cytology. The inclusion criteria were positive EGFR mutations, the diagnosis of BM, the use of TKIs, and WBRT. The exclusion criteria were a history of malignancies other than lung cancer, prior brain irradiation, or EGFR-TKI resistance mutation, or incomplete WBRT.

All the patients underwent pretreatment workups comprising a physical examination, a history review, chest radiography, bronchoscopy with a tumor biopsy, chest computed tomography (CT), brain magnetic resonance imaging (MRI) or CT, and routine laboratory studies. The tumor stage was classified according to the seventh edition of the American Joint Committee on Cancer (AJCC) Cancer Staging Manual and Handbook^23^. All patients started taking EGFR-TKIs once the diagnosis of stage IIIB or IV lung cancer with EGFR mutation was established. Some patients received BS which was performed prior to WBRT. BS was recommended at the discretion of neuro-surgeons after discussion with each patient. All patients with or without BS had WBRT. For WBRT, three-dimensional conventional radiotherapy was done by a 2100 C/D linear accelerator (Varian Medical Systems, Palo Alto, CA). Boost plans were generated by intensity-modulated radiotherapy either with an Eclipse, version 8.6 (Varian Medical Systems Inc., Palo Alto, USA) or Hi-Art helical tomotherapy unit, version 2.2.4.1 (TomoTherapy, Inc., Madison, WI). Our RT schedule was 30 Gy in 10 fractions or 37.5 Gy in 15 fractions. Some patients had a boost to BM of 45 Gy in 10 fractions, or 45 Gy in 15 fractions. The decision whether to boost BM was made after discussion with each patient.

The following variables were collected: age, sex, stage, initial clinical Tumor and Nodal classification, extracranial metastases, histological grading, smoking history, EGFR mutation, Eastern Cooperative Oncology Group (ECOG) performance status at the time of BM, number of BM, size of largest BM, whether the patient was symptomatic from BM, mean dose of ionizing radiation delivered, name of EGFR-TKI, number of lines of TKI, mean duration of TKI use, and number of lines of chemotherapy. The date of initial cancer diagnosis, the date of BM diagnosis, RT treatments, systemic therapy, most recent follow-up, and death were documented. In addition, a disease-specific Graded Prognostic Assessment (ds-GPA) was calculated for each patient to determine whether the cohorts shared similar prognostic features^24^.

### Statistical analysis

The primary end point was the survival after a diagnosis of BM was established. We calculated the survival from the date of BM diagnoses to the date of death from any cause or until the date of the last follow-up. And then we assessed the survival after a diagnosis of BM by Kaplan–Meier methods and used the log-rank test to compare time-to-event distributions. We stratified the data set and compared outcomes by *t*-test or chi-squared test. Besides, we performed univariate analyses and a multivariate Cox proportional hazards regression to examine all collected variables. We calculated the estimated risks of death using hazard ratios (HR) with 95% confidence intervals (CIs). The level of statistical significance was set at *P* < 0.05; all reported *P* values were two-tailed. The analyses used the SPSS software package, version 19.0 for Windows (SPSS, Chicago, IL, USA).

## Results

**Figure 1:**
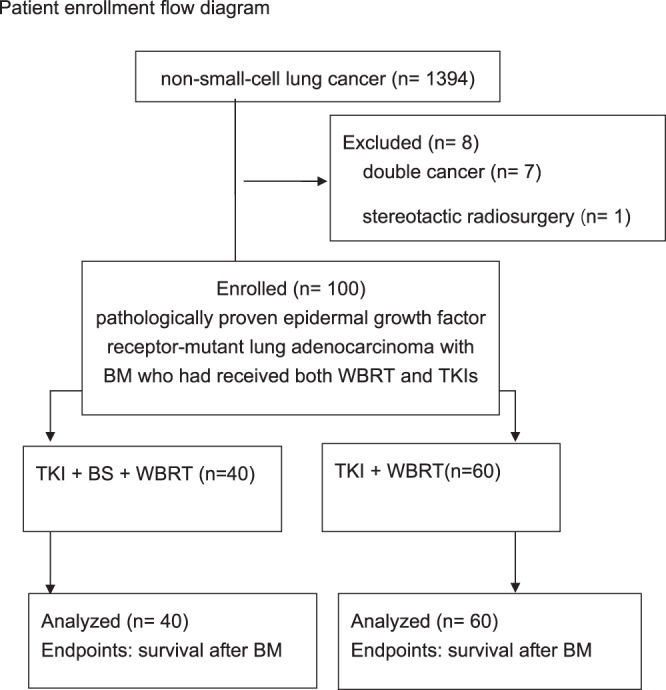
Patient enrollment flow diagram. Survival time was calculated from the date of BM diagnoses to the date of death or last follow-up. Abbreviations: BM: brain metastasis; WBRT: whole brain radiation therapy; TKI: tyrosine kinase inhibitor; BS: brain surgery.

There were 147 patients with EGFR-mutant NSCLC and BM, regardless of the treatment. One hundred patients out of 1394 patients in the lung cancer data base were identified after applying the aforementioned inclusion and exclusion criteria (Fig. 1). The clinical characteristics, divided by whether they had BS (TKI + BS + RT group vs TKI + RT group) were sum up in Table 1. All patients had both EGFR-TKI and WBRT. The mean age of this retrospective cohort was 60 ± 10 years ± standard deviation (SD), 96 patients (96%) had an ECOG performance status less than 2, and 78% were symptomatic from their BM. Forty patients (40%) received BS (TKI + BS + RT group), and 60 patients (60%) did not. Patients who received BS were more likely to have BM larger than 1 cm (90% in the TKI + BS + RT group and 60% in the TKI + RT group; *P* = 0.001). Patients who received BS were more likely to have EGFR mutation in exon 19 (60% in the TKI + BS + RT group and 36.7% in the TKI + RT group; *P* = 0.022) and were less likely to have EGFR mutation in exon 21 (27.5% in the TKI + BS + RT group and 48.3% in the TKI + RT group; *P* = 0.037). One patient has EGFR mutation in both exon 19 and 21. There was no significant difference in terms of age, gender, stage, initial clinical Tumor and Nodal classification, extracranial metastases, histological grading, smoking history, ECOG performance status at the time of BM, number of BM, whether the patient was symptomatic from BM, mean RT dose, number of lines of TKI, mean duration of TKI use, ds-GPA and number of lines of chemotherapy (all *P* > 0.05; Table 1).

**Table 1:** Patient and treatment characteristics.

	All	BS	No BS	*P*-value
No. of cases	100	40	60	
Mean Age (years ± SD)	60 ± 10	61 ± 9	60 ± 11	0.643
Sex				0.799
Female	64	25	39	
Male	36	15	21	
Initial Clinical stage				0.775
I-II	9	4	5	
III-IV	91	36	55	
Initial Tumor classification				0.78
1 or 2	26	11	15	
3 or 4	74	29	45	
Initial Nodal classification				0.239
0 or 1	38	18	20	
2 or 3	62	22	40	
Extracranial metastases				
Bone	72	26	46	0.203
Lung	38	15	23	0.933
Liver	15	3	12	0.086
Histological grade				0.119
1–2	40	15	25	
3	23	6	17	
NA	37	19	18	
EGFR mutation				
Exon 18	1	0	1	0.6 (Fisher)
Exon 19	46	24	22	0.022
Exon 20	8	5	3	0.164 (Fisher)
Exon 21	40	11	29	0.037
NA	6	1	5	0.397 (Fisher)
RT mean boost dose (cGy ± SD)	3779 ± 748	3908 ± 612	3694 ± 821	0.163
dose >3750 cGy	39	17	22	
dose ≦3750 cGy	61	23	38	
Number of lines of systemic chemotherapy				0.518 (Fisher)
0–3	89	37	52	
>3	11	3	8	
TKI name				
afatinib	12	8	4	0.044 (Fisher)
erlotinib	57	22	35	0.742
gefitinib	64	21	43	0.05
osimertinib	5	1	4	0.332 (Fisher)
Number of lines of TKI				0.447
1	63	27	36	
>1	37	13	24	
Mean TKI use duration (months ± SD)	19.2 ± 16.8	18 ± 14	20 ± 18.5	0.585
ECOG performance status				0.736
0	52	19	33	
1	44	19	25	
2	4	2	2	
Smoking status				0.182
Never	77	27	15	
Former	9	5	4	
Current	14	8	6	
Symptomatic BM				0.168
No	22	6	16	
Yes	78	34	44	
Size of the largest BM				0.001
≦1 cm	28	4	24	
>1 cm	72	36	36	
Number of BM				0.137
1	18	10	8	
>1	82	30	52	
dsGPA				0.373
0.5–1.5	70	26	44	
2–4	30	14	16	

Abbreviations: BS: brain surgery; EGFR: epidermal growth factor receptor; RT: radiation therapy; TKI: tyrosine kinase inhibitor; ECOG: Eastern Cooperative Oncology Group; BM: brain metastasis; dsGPA: disease-specific Graded Prognostic Assessment.

All patients started having EGFR-TKI (afatinib, erlotinib, gefitinib or osimertinib) once the diagnosis of stage IIIB or IV lung cancer with EGFR mutation was established. Twelve patients had afatinib; 57 patients had erlotinib; 64 patients had gefitinib; and 5 patients had osimertinib. Thirty-seven (37%) patients had more than one line of TKIs due to disease progression or intolerance of side effect. The median duration of TKIs use was 14.4 months (95% CI, 10.7 to 17.9). The median duration of TKIs use was 14 months (95% CI, 8.3 to 18.3) in the TKI + BS + RT group and 14.4 months (95% CI, 9.6 to 19) in the TKI + RT group. The mean duration of TKIs use were 18 ± 14 months and 20 ± 18.5 months for patients with and without BS respectively (*P* = 0.585).

After a median follow-up of 25.6 months (95% CI 18.6 to 35.7), the median survival after BM was 15.1 months (95% CI, 11.3 to 19.4) for the 100 patients in this study. The median survival after BM was 11.2 months (95% CI, 8.3 to 14.2) for the 147 patients irrespective of treatment. Specifically, the median survival after BM was 18.2 months (95% CI, 10.8 to 27.4) in the TKI + BS + RT group and 11.8 months (95% CI, 5.2 to18) in the TKI + RT group. The mean survival after BM were 21.9 ± 14.8 months and 15.6 ± 14.5 months for patients with and without BS respectively (*P* = 0.026).

**Figure 2:**
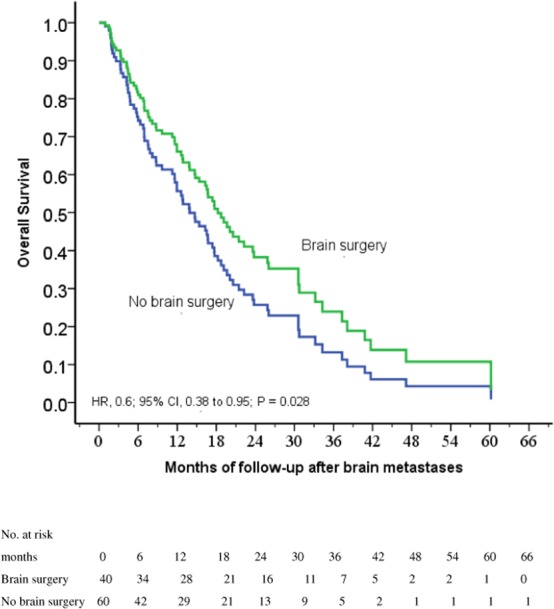
Cox regression comparing survival after the diagnosis of brain metastasis in epidermal growth factor receptor-mutant non-small-cell lung cancer patients under tyrosine kinase inhibitors treated with and without brain surgery for brain metastases before whole-brain radiation therapy.

Univariate analysis suggested that BS was a favorable prognostic factors for longer survival (hazard ratio HR, 0.6; 95% CI, 0.38 to 0.95; *P* = 0.028; Fig. 2). In addition, female (*P* = 0.005), exon 19 mutation (*P* = 0.048) and single BM relative to more than 3 BM (*P* = 0.01) were associated with improved OS (Table 1). However, after controlling for significant covariables in a multivariable model including gender, EGFR mutation in exon 19, and number of BM, the TKI + BS + RT group was not associated with improved OS relative to the TKI + RT group (HR, 0.69; 95% CI, 0.43 to 1.128; *P* = 0.134). EGFR mutation in exon 19 was not an independent favorable prognostic factor (HR, 0.83; 95% CI, 0.51 to 1.35; *P* = 0.461). Table 2 shows two independent favorable prognostic factors including female gender (HR, 0.56; 95% CI, 0.34 to 0.9; *P* = 0.017) and single BM relative to more than 3 BM (HR, 2.23; 95% CI, 1.11 to 4.45; *P* = 0.024).

**Table 2:** Univariate and multivariate Cox regression analyses of covariables associated with survival after brain metastases.

	Univariate analyses		Multivariate analyses	
	HR (95%CI)	*P*-value	HR (95%CI)	*P*-value
Age				
>65 vs. ≦65	1.35 (0.83 to 2.2)	0.226		
Female vs. male	0.5 (0.31 to 0.79)	0.005	0.56 (0.34 to 0.9)	**0.017**
Initial Clinical stage				
III–IV vs. I–II	1.25 (0.54 to 2.88)	0.6		
Initial Tumor classification				
III–IV vs. I–II	1.39 (0.83 to 2.33)	0.208		
Initial Nodal classification				
2–3 vs. 0–1	1.48 (0.93 to 2.35)	0.097		
EGFR mutation				
Exon 19 or 21				
Yes vs. no	0.69 (0.38 to 1.26)	0.225		
Exon 19				
Yes vs. no	0.63 (0.4 to 1)	0.048	0.83 (0.51 to 1.35)	0.461
Exon 20				
Yes vs. no	0.95 (0.44 to 2.08)	0.905		
Exon 21				
Yes vs. no	1.18 (0.75 to 1.85)	0.47		
Brain surgery				
Yes vs. no	0.6 (0.38 to 0.95)	0.028	0.69 (0.43 to 1.12)	0.134
RT boost dose >3750 cGy				
Yes vs. no	0.84 (0.53 to 1.32)	0.441		
Number of lines of systemic chemotherapy				
>3 vs. 0–3	1.26 (0.63 to 2.53)	0.519		
TKI name				
afatinib				
Yes vs. no	0.56 (0.26 to 1.22)	0.144		
erlotinib				
Yes vs. no	0.72 (0.46 to 1.12)	0.141		
gefitinib				
Yes vs. no	1.43 (0.89 to 2.31)	0.142		
osimertinib				
Yes vs. no	0.68 (0.21 to 2.15)	0.505		
Number of lines of TKI				
>1 vs. 1	0.8 (0.5 to 1.26)	0.326		
ECOG performance status				
1 vs. 0	1.03 (0.66 to 1.61)	0.885		
2 vs. 0	0.46 (0.11 to 1.91)	0.287		
Smoking status				
Former or current vs. never	1.39 (0.84 to 2.3)	0.206		
Symptomatic brain metastases				
Yes vs. no	1.13 (0.65 to 1.95)	0.671		
Size of the largest brain tumor				
>1 cm vs. ≦1 cm	1.51 (0.9 to 2.53)	0.121		
No. of brain metastases				
2–3 vs. 1	2.11 (0.95 to 4.93)	0.068	1.83 (0.81 to 4.14)	0.149
>3 vs. 1	2.45 (1.24 to 4.72)	0.01	2.23 (1.11 to 4.45)	**0.024**
dsGPA				
0.5–1.5 vs. 2–4	1.6 (0.96 to 2.65)	0.07		

Abbreviations: EGFR: epidermal growth factor receptor; RT: radiation therapy; TKI: tyrosine kinase inhibitor; ECOG: Eastern Cooperative Oncology Group; dsGPA: disease-specific Graded Prognostic Assessment.

### BM size

**Figure 3:**
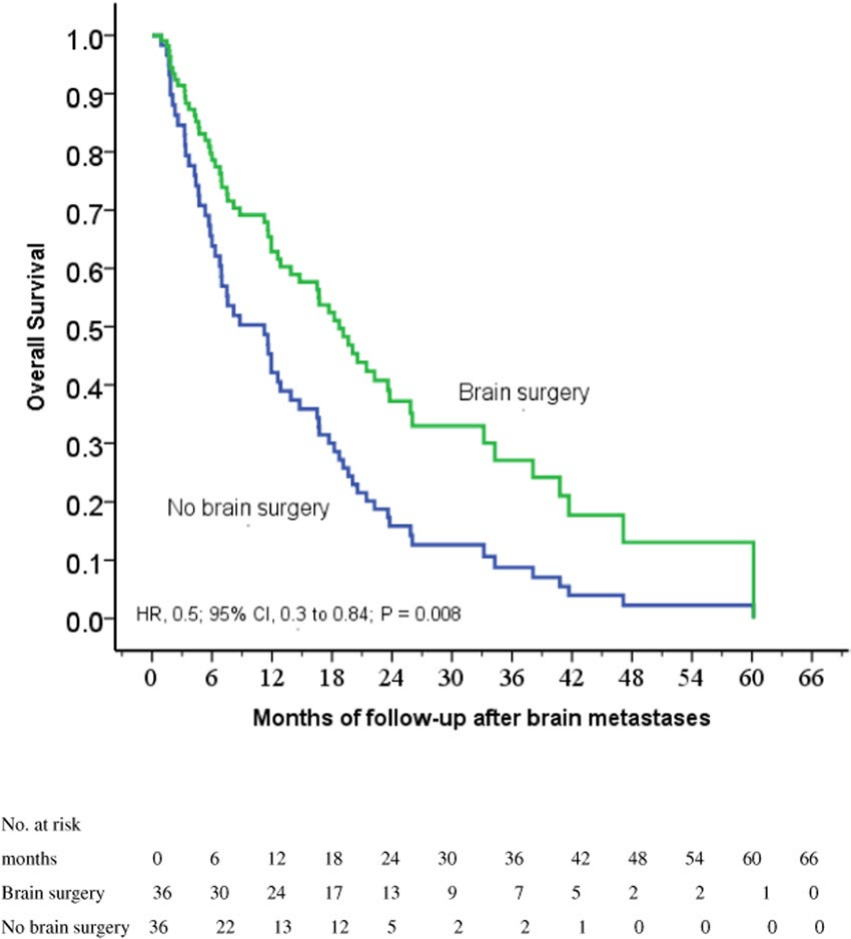
Cox regression comparing survival after the diagnosis of brain metastasis in epidermal growth factor receptor-mutant non-small-cell lung cancer patients under tyrosine kinase inhibitors with a size of the largest brain metastasis over 1 cm treated with and without brain surgery for brain metastases before whole-brain radiation therapy.

In order to identify potential differences in the benefits of BS in patients by the size of largest BM, we selected 72 patients with the largest BM larger than 1 cm. Thirty-six (50%) patients had BS. Cox regression analysis revealed that BS was a strong favorable prognostic factor for longer survival (HR, 0.5; 95% CI, 0.3 to 0.84; *P* = 0.008; Fig. 3). In Table 3, after controlling for significant covariables in a multivariable model, the TKI + BS + RT group was associated with improved OS relative to the TKI + RT group (HR, 0.49; 95% CI, 0.29 to 0.83; *P* = 0.008). Clinical nodal classification 0–1 relative to 2–3 (HR, 2.23; 95% CI, 1.27 to 3.92; *P* = 0.005) and the use of erlotinib (HR, 0.49; 95% CI, 0.29 to 0.85; *P* = 0.011) were also beneficial.

**Table 3:** For patients with the largest brain metastasis over 1 cm: univariate and multivariate Cox regression analyses of covariables associated with survival after the diagnosis of brain metastasis.

	Univariate analyses		Multivariate analyses	
	HR (95%CI)	*P*-value	HR (95%CI)	*P*-value
Age				
>65 vs. ≦65	1.32 (0.77 to 2.29)	0.318		
Female vs. male	0.59 (0.35 to 1)	0.052		
Initial Clinical stage				
III–IV vs. I–II	1.38 (0.5 to 3.81)	0.538		
Initial Tumor classification				
III–IV vs. I–II	0.92 (0.5 to 1.67)	0.772		
Initial Nodal classification				
2–3 vs. 0–1	1.67 (0.98 to 2.83)	0.058	2.23 (1.27 to 3.92)	**0.005**
EGFR mutation				
Exon 19 or 21				
Yes vs. no	0.815 (0.43 to 1.55)	0.533		
Exon 19				
Yes vs. no	0.68 (0.41 to 1.15)	0.15		
Exon 20				
Yes vs. no	0.82 (0.372 to 1.81)	0.622		
Exon 21				
Yes vs. no	1.12 (0.65 to 1.9)	0.687		
Brain surgery				
Yes vs. no	0.5 (0.3 to 0.84)	**0.008**	0.49 (0.29 to 0.83)	**0.008**
RT boost dose >3750cGy				
Yes vs. no	1.1 (0.65 to 1.85)	0.726		
Number of lines of systemic chemotherapy				
>3 vs. 0–3	1.18 (0.53 to 2.61)	0.684		
TKI name				
afatinib				
Yes vs. no	1.16 (0.46 to 2.9)	0.757		
erlotinib				
Yes vs. no	0.57 (0.34 to 0.95)	0.031	0.49 (0.29 to 0.85)	**0.011**
gefitinib				
Yes vs. no	1.43 (0.83 to 2.48)	0.199		
osimertinib				
Yes vs. no	1.31 (0.41 to 4.19)	0.655		
Number of lines of TKI				
>1 vs. 1	0.692 (0.4 to 1.2)	0.192		
ECOG performance status				
1 vs. 0	0.96 (0.57 to 1.62)	0.889		
2 vs. 0	0.59 (0.14 to 2.47)	0.467		
Smoking status				
Former or current vs. never	1.25 (0.71 to 2.2)	0.434		
Symptomatic brain metastases				
Yes vs. no	0.73 (0.31 to 1.71)	0.471		
No. of brain metastases				
2–3 vs. 1	1.62 (0.75 to 3.49)	0.216		
>3 vs. 1	1.4 (0.58 to 3.39)	0.451		
dsGPA				
0.5–1.5 vs. 2–4	1.38 (0.74 to 2.55)	0.311		

Abbreviations: EGFR: epidermal growth factor receptor; RT: radiation therapy; TKI: tyrosine kinase inhibitor; ECOG: Eastern Cooperative Oncology Group; dsGPA: disease-specific Graded Prognostic Assessment.

### EGFR mutation

In order to identify potential differences in the benefits of BS in patients with EGFR mutation, we selected 85 patients with mutation in exon 19 or 21 or both. Thirty-four (40%) patients had BS. Cox regression analysis revealed that female, BS and single BM were favorable prognostic factors for longer survival (Table 4). However, after controlling for significant covariables in a multivariable model, the TKI + BS + RT group was not associated with improved OS relative to the TKI + RT group (HR, 0.66; 95% CI, 0.4 to 1.11; *P* = 0.116). Female (HR, 0.47; 95% CI, 0.28 to 0.78; *P* = 0.004) and single BM relative to more than 3 BM (HR, 2.41; 95% CI, 1.16 to 5; *P* = 0.018) were two independent favorable prognostic factors.

**Table 4:** For patients with EGFR mutation in exon 19 or exon 21: univariate and multivariate Cox regression analyses of covariables associated with survival after the diagnosis of brain metastasis.

	Univariate analyses		Multivariate analyses	
	HR (95%CI)	*P*-value	HR (95%CI)	*P*-value
Age				
>65 vs. ≦65	1.32 (0.78 to 2.24)	0.302		
Female vs. male	0.46 (0.28 to 0.76)	0.003	0.47 (0.28 to 0.78)	**0.004**
Initial Clinical stage				
III–IV vs. I–II	2.57 (0.63 to 10.51)	0.19		
Initial Tumor classification				
III–IV vs. I–II	1.43 (0.81 to 2.51)	0.215		
Initial Nodal classification				
2–3 vs. 0–1	1.53 (0.91 to 2.56)	0.105		
Brain surgery				
Yes vs. no	0.59 (0.36 to 0.97)	0.036	0.66 (0.4 to 1.11)	0.116
RT boost dose >3750cGy				
Yes vs. no	0.81 (0.48 to 1.35)	0.41		
Number of lines of systemic chemotherapy				
>3 vs. 0–3	1.96 (0.93 to 4.15)	0.077		
TKI name				
afatinib				
Yes vs. no	0.45 (0.18 to 1.12)	0.86		
erlotinib				
Yes vs. no	0.82 (0.51 to 1.33)	0.427		
gefitinib				
Yes vs. no	1.49 (0.86 to 2.6)	0.159		
osimertinib				
Yes vs. no	0.65 (0.16 to 2.65)	0.543		
Number of lines of TKI				
>1 vs. 1	0.938 (0.57 to 1.54)	0.798		
ECOG performance status				
1 vs. 0	1.05 (0.65 to 1.71)	0.842		
2 vs. 0	0.34 (0.05 to 2.46)	0.283		
Smoking status				
Former or current vs. never	1.43 (0.81 to 2.54)	0.216		
Symptomatic brain metastases				
Yes vs. no	1.13 (0.61 to 2.07)	0.697		
Size of largest brain tumor				
>1 cm vs ≦1 cm	1.47 (0.85 to 2.53)	0.168		
No. of brain metastases				
2–3 vs. 1	2.03 (0.84 to 4.91)	0.118	1.8 (0.74 to 4.4)	0.198
>3 vs. 1	2.57 (1.25 to 5.28)	0.01	2.41 (1.16 to 5)	**0.018**
dsGPA				
0.5–1.5 vs. 2–4	1.58 (0.92 to 2.71)	0.098		

Abbreviations: EGFR: epidermal growth factor receptor; RT: radiation therapy; TKI: tyrosine kinase inhibitor; ECOG: Eastern Cooperative Oncology Group; dsGPA: disease-specific Graded Prognostic Assessment.

## Discussion

The mainstay of treatment for BM consisted of surgical resection, RT, or a combination of these modalities. A prospective randomized study showed that patients with cancer and a single BM who received BS plus RT lived longer^25^. A further study showed that BS followed by consolidative WBRT was better than BS alone for local control^26^. RT is commonly used following BS since local recurrence occurs in more than 50% of patients^27^. In two randomized trials, postoperative adjuvant WBRT reduced the incidence of local recurrence by half^27,28^. Twenty years ago, additional postoperative WBRT with 30Gy for patients with single BM was reported (BS + WBRT: median OS 13 months; BS only: median OS 8 months). In addition, the rate of cerebral recurrence was distinctly higher in the non-WBRT group^29^.

Toffart *et al*. concluded that the survival of NSCLC with synchronous solitary M1 was more similar to stage III than other stage IV NSCLC and advocated for BS^30^. In this large retrospective study of 4832 patients, 64% of patients had BM. Operation conducted at both primary and metastatic sites (HR 0.35, 95% CI: 0.19 to 0.65) was an independent prognostic factor for longer survival. For accessible tumors with diameter of more than 3 cm, BS still carries the advantages of obtaining histological diagnosis, providing immediate symptomatic relief by removal of local mass effect and source of edema, and decreasing the length of steroid use^21^.

At the time of the studies of Patchell *et al*., however, EGFR-TKIs were not available^25,28^. In the era of EGFR mutations and TKIs, we seek to compare survival trends that are likely to be attributable to combined treatment, especially BS plus TKIs and WBRT. We investigated one hundred patients who received TKIs as a first-line therapy for advanced EGFR-mutant NSCLC in a tertiary cancer center. Because the administration of EGFR-TKI has limited penetration across BBB, combined RT would provide better outcome^31^. According to Burel-Vandenbos *et al*., BM occurring during the course of TKI, despite good control of extracranial disease, is possibly due to insufficient concentration of TKI in cerebral spinal fluid^32^ although raising TKI doses might increase the possibility of drug intolerance. Soon *et al*. conducted a meta-analysis from 2008 to July 2014, and reported there was a better 2-year OS (HR 1.33, 95% CI 1.00–1.77; *P* = 0.05) for patients with upfront WBRT compared with TKI alone^33^.

However, NSCLC is a radioresistant malignancy and 30 Gy of WBRT may not be sufficient to sterilize the metastatic brain lesions^32^, therefore combination therapy is required. We hypothesized that BS would be beneficial prior to WBRT. After controlling for significant covariables in a multivariable model, EGFR mutation in exon 19 (HR, 0.83; 95% CI, 0.51 to 1.35; *P* = 0.461) or the TKI + BS + RT group was not associated with improved survival relative to the TKI + RT group (HR, 0.69; 95% CI, 0.43 to 1.12; *P* = 0.134). However, for the 72 patients with the largest BM over 1 cm, multivariate analysis showed that the TKI + BS + RT group was associated with improved survival relative to the TKI + RT group (HR, 0.49; 95% CI, 0.29 to 0.83; *P* = 0.008). For the majority of the patients with EGFR mutation in exon 19 or 21, Female and single BM relative to more than 3 BM were two strong independent favorable prognostic factors.

The present study demonstrated the advantage of BS, especially for patients with stage IV EGFR-mutant NSCLC who had the largest BM over 1 cm. To the best of our knowledge, this is the first study to confirm the advantage from combination therapy of TKI + BS + RT. Until recently, there was no prospective randomized trial regarding the addition of BS to TKIs and WBRT.

The limitations of the current study are the inherent biases in retrospective studies. The pitfalls include limited patient numbers, possible selection bias from surgical intervention, incomplete records of post-operative complications, cognitive evaluation and intracranial control. This study enrolled a real-world population of NSCLC patients, including sicker patients who were not eligible for a clinical trial. Although the patients were not randomized, fundamentally, similar characteristics existed between TKI + BS + RT and TKI + RT groups. In the current study, we focused on prognostic features, molecular markers, and survival change from the addition of BS to the combination of TKIs and WBRT.

## Conclusions

BS prior to WBRT adds significant survival benefits in addition to the combination of TKIs and WBRT, especially for patients with EGFR-mutant NSCLC who had the largest BM over 1 cm. This observational study on BS outcome needs to be interpreted with some caution because there are potential confounding factors. The result should be cautiously applied. Further prospective study is warranted.

## Data Availability

The data used to support the findings of this study are included within the article.
